# Revisiting rodent models: *Octodon degus* as Alzheimer’s disease model?

**DOI:** 10.1186/s40478-016-0363-y

**Published:** 2016-08-26

**Authors:** Johannes Steffen, Markus Krohn, Kristin Paarmann, Christina Schwitlick, Thomas Brüning, Rita Marreiros, Andreas Müller-Schiffmann, Carsten Korth, Katharina Braun, Jens Pahnke

**Affiliations:** 1 University of Lübeck (UzL), LIED, Lübeck, Germany; 2 Department of Neuro-/Pathology, University of Oslo (UiO) & Oslo University Hospital (OUS), Oslo, Norway; 3 Department of Neuropathology, Heinrich Heine University Düsseldorf (HHU), Düsseldorf, Germany; 4 Department of Zoology/Developmental Neurobiology, Otto von Guericke University Magdeburg (OvGU), Magdeburg, Germany; 5 Leibniz Institute for Plant Biochemistry (IPB), Halle, Germany; 6 Department of Pathology (PAT), Translational Neurodegeneration Research and Neuropathology Lab, University of Oslo, Postboks 4950, Nydalen, 0424 Oslo Norway

**Keywords:** Neurodegenerative diseases, Neuropathology, Rodentia, Amyloid beta-Peptides, Tau proteins, Alzheimer’s disease, Animal models, *Octodon degus*

## Abstract

Alzheimer’s disease primarily occurs as sporadic disease and is accompanied with vast socio-economic problems. The mandatory basic research relies on robust and reliable disease models to overcome increasing incidence and emerging social challenges. Rodent models are most efficient, versatile, and predominantly used in research. However, only highly artificial and mostly genetically modified models are available. As these ‘engineered’ models reproduce only isolated features, researchers demand more suitable models of sporadic neurodegenerative diseases. One very promising animal model was the South American rodent *Octodon degus*, which was repeatedly described as natural ‘sporadic Alzheimer’s disease model’ with ‘Alzheimer’s disease-like neuropathology’. To unveil advantages over the ‘artificial’ mouse models, we re-evaluated the age-dependent, neurohistological changes in young and aged *Octodon degus* (1 to 5-years-old) bred in a wild-type colony in Germany. In our hands, extensive neuropathological analyses of young and aged animals revealed normal age-related cortical changes without obvious signs for extensive degeneration as seen in patients with dementia. Neither significant neuronal loss nor enhanced microglial activation were observed in aged animals. Silver impregnation methods, conventional, and immunohistological stains as well as biochemical fractionations revealed neither amyloid accumulation nor tangle formation. Phosphoepitope-specific antibodies against tau species displayed similar intraneuronal reactivity in both, young and aged *Octodon degus*.

In contrast to previous results, our study suggests that *Octodon degus* born and bred in captivity do not inevitably develop cortical amyloidosis, tangle formation or neuronal loss as seen in Alzheimer’s disease patients or transgenic disease models.

## Introduction

Senile plaques, a hallmark of Alzheimer’s disease (AD), were long suggested to initiate the destructive cascade to progressive neuronal dysfunction and death. Nowadays small, soluble oligomers of β-amyloid (Aβ) are deemed the primary toxic species [1]. These oligomers disrupt a variety of receptors [2], increase membrane permeability [2] and are suspected to induce hyperphosphorylation and aggregation of tau [3]. Physiologically, Aβ is largely eliminated from the brain by LRP1 [4] and several ABC transporters (reviewed in [5, 6]). The vast majority of cases occur sporadically and a large series of risk factors have been identified, including age, type 2 diabetes, high blood pressure, and various genetic factors like specific alleles of apolipoprotein E (APOE) [7, 8].

A small proportion of AD cases involve genetic variations which entail alterations in amount, ratio or amino acid sequence of Aβ [9]. However, these rare inherited forms are the fundament of both, disease models and our current understanding of AD. Due to a lack of alternatives, the main focus lies on the usage of these genetically manipulated research animals (thus non-sporadic AD models), which restricts the progress of research and limits the scope of detailed analyses. To successfully combat the sporadic form of AD, models that develop the disease on a more ‘natural’ basis would certainly help to understand the underlying mechanisms which are essential for developing advanced and efficient therapy options.

**Fig. 1:**
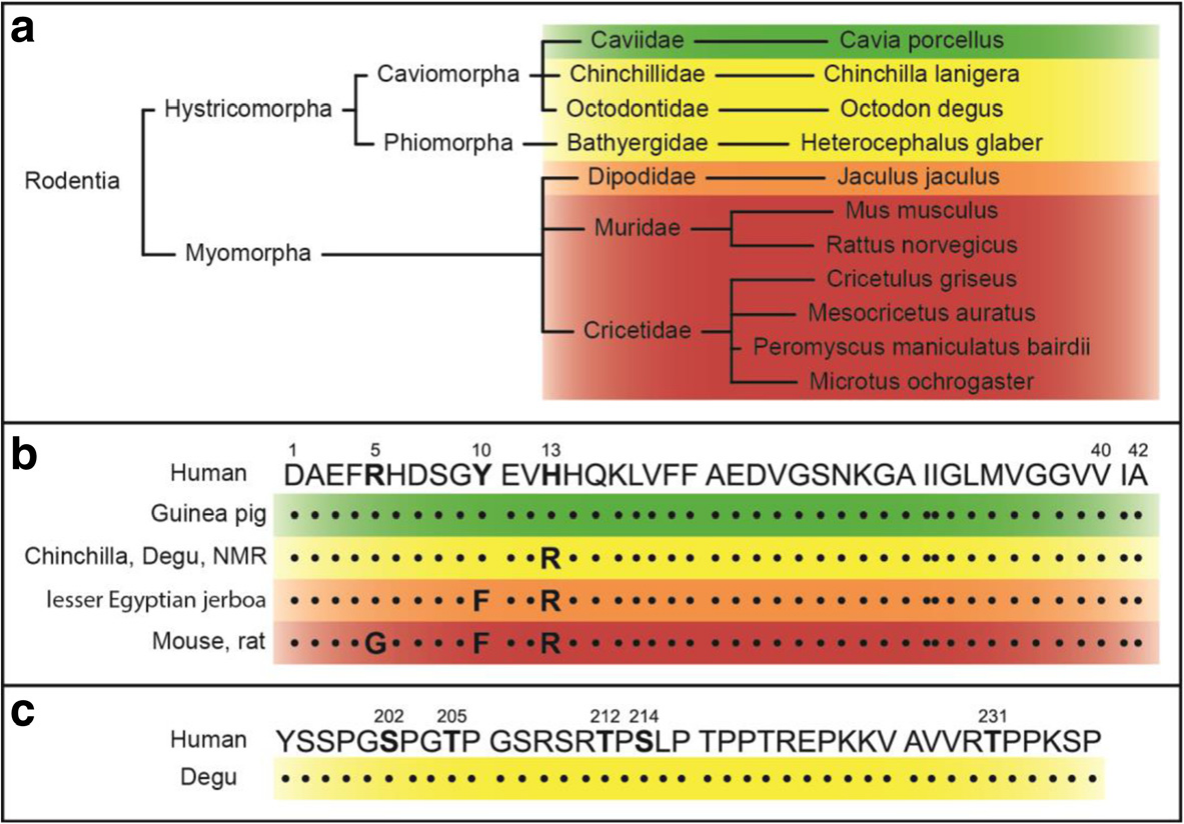
Comparison of human and rodent Aβ sequences. Part **a** shows a simplified phylogenetic tree of rodents. In **b**, human and rodent sequences of Aβ_42_ are compared. The Aβ sequence of guinea pigs (*green*) equals the human sequence. Chinchillas, degus and naked mole rats (NMRs) share the same sequence (*yellow*) with one variation at position 13 as compared to humans. The lesser Egyptian jerboa has an additional difference at position 10 (*orange*). A larger group of rodents, including mice and rats, show 3 sequential differences at 5, 10 and 13 (*red*). This sequence is often erroneously referred to as “rodent Aβ”, as it is the most frequent sequence in rodents. The uniformity of human and degu tau at the analysed phosphorylation sites is shown in **c**

The South American rodent *Octodon degus* (degu) may be a promising candidate for physiologically modelling sporadic AD, as it was reported to develop the ‘*full range of AD-like pathologies*’ [10] without any genetic manipulation. While wild degus have only a limited life expectancy (mean: <1 year; common max: 3–4 years), captivity lowers mortality and increases mean life span to 5–8 years [11] (Ebensperger L.A. & Hayes L.D., unpublished data). This captivity-dependent, aged phenotype combined with the highly homologous Aβ sequence [12], differing only in one amino acid from human Aβ (see Fig. 1b), might be the main reasons for their vulnerability. Thus, during the past years wild-caught and captive born degus were used in AD research [12–15] and have been referenced in numerous review publications [10, 16–22]. In this course, prominent intra- and extracellular Aβ deposits were reported in cortical and hippocampal areas of aged animals (>3 years) [12, 19]. Furthermore, APP and Aβ positive axonal bulbs were observed in hippocampal white matter tracts of old animals (6 years) which preceded cerebral amyloid angiopathy [13]. Biochemical analysis of aged degus (5-years-old) indicated a correlation between Aβ*56 oligomers and tau phosphorylation on the one hand and decreased synaptic plasticity and impaired memory performance on the other hand [14]. However, the most recent study, analysing young (1 to 3 years old) and old animals (4 to 6 years old), found AD associated autophagy markers LC3 and p62 unchanged [15]. Furthermore, GFAP (glial fibrillary acidic protein), CD11b expression, oxidative stress and apoptosis markers were unaffected in cortices but elevated in hippocampi of old animals. Cortical and hippocampal levels of AD-linked IL-6 seemed increased in old degus [15].

The aim of the present study was to critically re-evaluate the suitability of degus as ‘natural’ AD model. To characterize amyloidosis and tau deposition, different cortical and hippocampal regions of young (1-year-old) and aged (5-years-old) wild-type, colony-bred degus were screened for neurodegenerative changes.

## Materials and methods

Drugs and chemicals used in the study were purchased from Carl Roth, Karlsruhe, Germany; except for those specifically mentioned.

### Animals

Wild-type degus used in the present study were bred at the Institute of Biology, Otto-von-Guericke-University Magdeburg. Degus were housed in a climate-controlled environment (22 °C, 55 ± 5 % humidity) on a 12 h light/dark cycle with an enriched environment. Social enrichment was provided by housing in same sex groups of up to 4 animals. Sensory, motor and cognitive enrichment was provided by housing the animals in large cages (510x420x680 mm^3^, EBECO, Castrop Rauxel, Germany), equipped with a drinking bottle, burrows for hiding, a running wheel (Europet-Bernina International, Iserlohn, Germany) for physical exercise and material for nest building. Nutritional enrichment was provided by providing dry bread, fresh vegetables and fresh tree branches in addition to the regular pellet food (rat diet pellets and cereals; ssniff Spezialdiäten, Soest, Germany) ad libitum. A first cohort of degus was immunohistochemically analysed (J.P.) in two groups: young (12 months) and aged (60 months), both sexes, at least two animals per sex and age group. A second cohort from the same colony was independently analysed in another neuropathological laboratory (C.K.) and used for quantitative and western blot analyses: seven animals (age in months: 3, 24, 25, 56, 56, 65, and 65)

Tg-mice, harbouring mutant human amyloid precursor protein (KM670/671NL) and presenilin 1 (L166P) both driven by murine Thy1.2-promotor [23], were kindly provided by the University of Tübingen, Germany. All mice were housed in a climate-controlled (22 °C) environment on a 12 h light/dark cycle in same sex groups of up to 4 animals and free access to food and water. All experiments were conducted in accordance to the EU and state law of Saxony-Anhalt and approved by the local animal ethics committee.

### Sequences & alignments

Protein sequences were gathered using NCBI Protein database (http://www.ncbi.nlm.nih.gov/protein; for accession numbers see Table 1) and protein alignments were performed using BLASTP 2.2.30+ [24, 25].

**Table 1:** Accession numbers of featured proteins from the NCBI Protein database (http://www.ncbi.nlm.nih.gov/protein/)

Species	Accession number
Amyloid-beta A4 protein isoform a precursor	
Homo sapiens	NP_000475.1
Cavia porcellus	XP_003467233.1
Chinchilla lanigera	XP_005375649.1
Octodon degus	XP_004627753.1
Heterocephalus glaber	XP_004898345.1
Jaculus jaculus	XP_004654437.1
Mus musculus	NP_001185752.1
Rattus norvegicus	NP_062161.1
Cricetulus griseus	ERE75573.1
Mesocricetus auratus	XP_005073973.1
Peromyscus maniculatus	XP_006988006.1
Microtus ochrogaster	XP_005345348.1
Microtubule-associated protein tau	
Homo sapiens	NP_001116538.2
Octodon degus	XP_004630049

### Immunohistochemistry

Animals were sacrificed by cervical dislocation and immediately perfused with 50 mL PBS followed by 50 mL PFA for fixation. Paraffin-embedded, 4 μm-thick coronal sections were deparaffinised and stained using Haematoxylin and Eosin. Immunohistochemical analyses were performed using Bond-Max™ (Leica Microsystems, Wetzlar, Germany) automated staining system as described previously [5, 26–28]. Epitope retrieval was carried out as follows: 5 min in 95 % (v/v) formic acid for 6F3D, 4G8 and 6E10; 20 min in EDTA buffer pH 9.0 for IBA1 and AT180; 10 min enzymatic digestion (Bond Enzyme Pretreatment Kit, Leica Biosystems Nussloch, Nussloch, Germany) for GFAP or 20 min in citric acid buffer pH 6.0 for NeuN, AT8 and AT100. Antibodies against ionized calcium-binding adapter molecule 1 (IBA1 1:1000, 019-19741, Wako Chemicals, Neuss, Germany), glial fibrillary acid protein (GFAP, 1:500, Z033401, Dako Deutschland, Hamburg, Germany), neuronal nuclear antigen (NeuN, 1:500, MAB377, Merck Chemicals, Darmstadt, Germany) phosphorylated tau (AT8, 1:50, MN1020; AT100, 1:500, MN1060; AT180, 1:50, MN1040, Thermo Fisher Scientific, Waltham, MA, USA); β-amyloid (6E10, 1:100, SIG-39320, Covance Research Products, Denver. PA, USA; 4G8, 1:2000, SIG-39220, Covance Research Products, Denver. PA, USA; 6F3D, 1:100, M0872, Dako Deutschland, Hamburg, Germany) were used according to manufactures instructions. Slides were developed using Bond™ Polymer Refine Detection kit (Leica Microsystems, Wetzlar, Germany). For Campbell-Switzer staining, paraffin-embedded, 4 μm-thick coronal sections were deparaffinised, stirred for 5 min in ammonium hydroxide and washed twice in distilled water for 1 min. Sections were incubated for 40 min in Silver-Pyridine-Carbonate solution (14 % (v/v) pyridine, 0.49 % (w/v) silver nitrate, 0.37 % (w/v) potassium carbonate) followed by 3 min in citric acid and washed in acetate buffer (33.6 mM sodium acetate, 14.4 mM acetic acid, pH 4.99). Sections were developed in developer solution (236 mM sodium carbonate, 12.5 mM ammonium nitrate, 5.9 mM silver nitrate, 1.7 mM tungstosilicic acid, 0.87 mM formaldehyde) under a light source for about 6 min. Sections were washed three times in acetate buffer and once in distilled water, each for 30 s. Sections were finally placed in 0.5 % (w/v) sodium thiosulfate solution for 45 s, washed twice in distilled water and subsequently mounted. Slides were digitized using MIRAX MIDI Scanner (Carl Zeiss MicroImaging, Jena, Germany). The scanned slides were processed using the free Pannoramic Viewer software (3DHISTECH, Budapest, Hungary), and analysed under blinded conditions using either AxioVision (Carl Zeiss Imaging Solutions; Munich, Germany) or ImageJ (Wayne Rasband, National Institutes of Health, Bathesda, MD, USA) and the ITCN plugin (Thomas Kuo and Jiyun Byun, University of California, CA, USA).

### Four-step fractionation and quantification of Aβ

Animals were sacrificed by cervical dislocation and transcardially perfused with PBS. The brain was immediately snap-frozen in liquid nitrogen and stored at −80 °C. Fractionation of brain tissue was performed by preparative ultracentrifugation as described previously [29]. In brief, brain tissue was homogenated in 9 volumes of TBS buffer (150 mM sodium chloride, 50 mM Tris, pH 7, supplemented with protease inhibitor, Complete EDTA-free, Roche, Basel, Switzerland) and subsequently centrifuged (100,000 g, 4 °C, 1 h). The supernatant (TBS-fraction, soluble Aβ) was harvested, pellet was sonicated in 100 μl TBS/ 1 % (v/v) Triton X-100 and centrifuged again (100,000 g, 4 °C, 1 h). The supernatant (TX-100-fraction, detergent soluble Aβ) was harvested, the pellet was sonicated in TBS/ 2 % (w/v) SDS and centrifuged (100,000 g, room temperature, 1 h). Supernatant (SDS-fraction, protein bound Aβ) was harvested and the remaining pellet was finally resolved in 70 % formic acid (FA-fraction, insoluble Aβ). For quantification, SDS-fraction was diluted 20-fold in TBS and FA-fraction was neutralized with 19 volumes of 1 M Tris (pH 11).

Quantification was performed using the Human/Rat β-amyloid (40) or (42) ELISA Kit (Wako Chemicals, Neuss, Germany) according to the manufacturer's instructions, which uses BNT77 (epitope: amino acids 11-16) and BA27 (Aβ_40_-specific) or BC05 (Aβ_42_-specific), respectively [30].

### Tau preparation

Brain tissue was mixed with 9 volumes of TBS buffer (supplemented with protease and phosphatase inhibitor, Sigma-Aldrich, St. Louis, MO, USA), homogenized (total fraction) and centrifuged (130,000 g, 4 °C, 20 min). The pellet was resuspended in RIPA buffer (150 mM sodium chloride, 50 mM Tris, 0.5 % (w/v) deoxycholic acid, 1 % (v/v) Triton X-100, 0.5 % (w/v) SDS, 25 mM EDTA, pH 8 supplemented with protease and phosphatase inhibitor) and centrifuged again (130,000 g, 4 °C, 20 min). Supernatant was discarded; pellet was resuspended in 70 % formic acid and centrifuged (130,000 g, 4 °C, 20 min). Supernatant (FA-fraction) was neutralized with two volumes of neutralization buffer (5 M sodium hydroxide, 0.5 M Tris, 0.25 M monosodium phosphate). Proteins were precipitated using 2,2,2-trichloroacetic acid (25 % (v/v) TCA) for 30 min at 4 °C and subsequently separated by centrifugation (22,000 g, 4 °C, 15 min). Resulting pellet (insoluble fraction) was washed two times with cold acetone and subsequently air-dried.

### Western blot

Total and insoluble fractions were separated on a reducing 4–12 % Bis-Tris gel (Thermo Fisher Scientific, Waltham, MA, USA) and subsequently transferred to nitrocellulose membrane. Phosphorylated tau was detected using AT8 (1:1,000, MN1020, Thermo Fisher Scientific, Waltham, MA, USA) and total tau levels using HT7 antibody (1:1,000, MN1000, Thermo Fisher Scientific, Waltham, MA, USA), and protein expression was normalized with internal control anti-actin (1:10,000, Sigma-Aldrich, St. Louis, MO, USA).

### Statistics

For all quantifications, animals of either sex were used (n ≥ 2 per sex; n ≥ 4 per group), statistical significance (*p* ≤ 0.05) was determined using unpaired t-tests with Welch’s correction (Prism 6, GraphPad Software La Jolla, CA, USA).

## Results

**Fig. 2:**
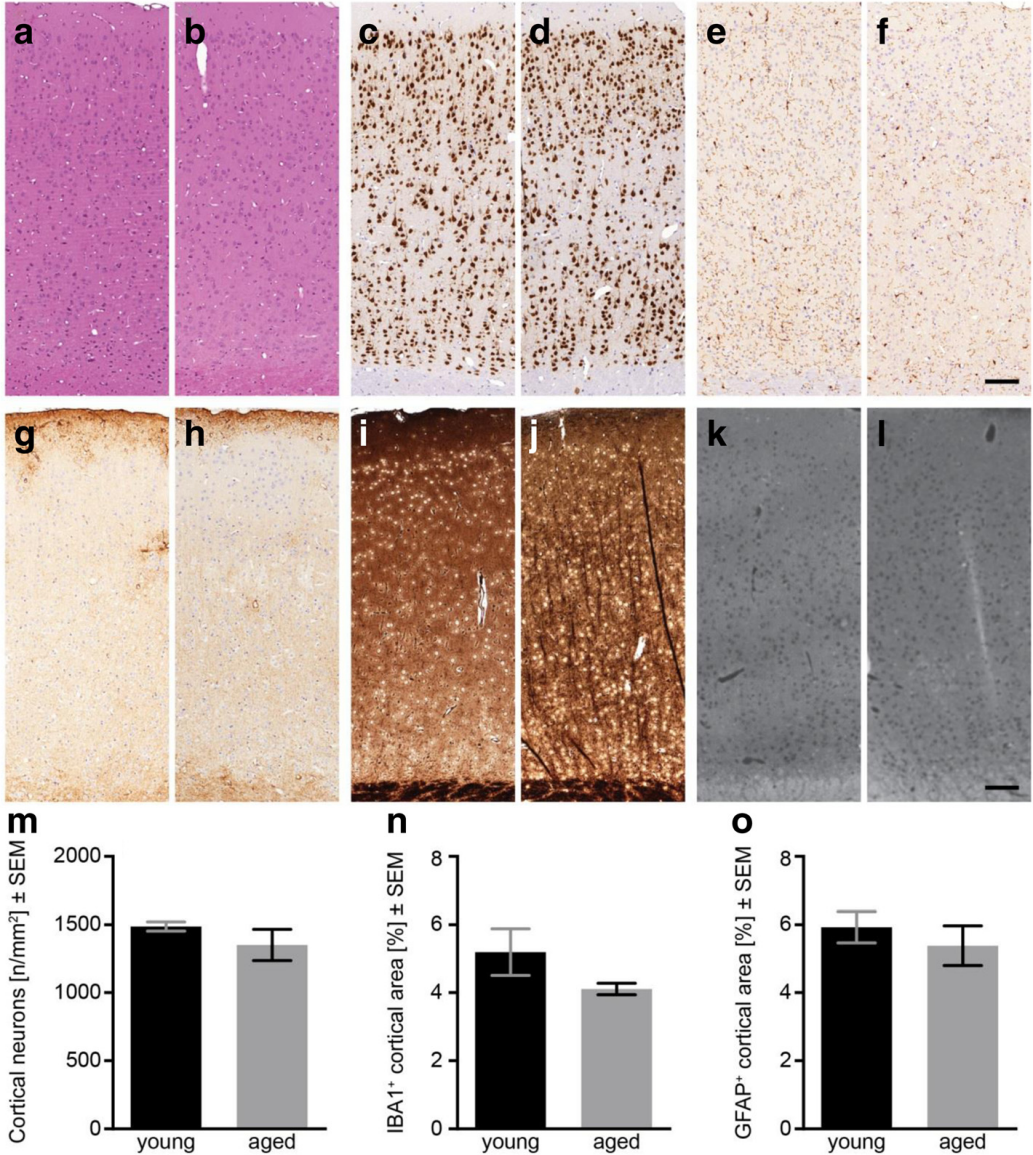
Immunohistochemical analysis of young and aged degus. H&E stain revealed normal age-related changes but no signs for lesions, neurodegeneration, or displacement in young (1-year-old, **a**) and aged (5-years-old, **b**) animals. Density of cortical neurons (NeuN-satin) remained virtually unchanged in aged degus (**d**), compared to young (**c**). IBA1-stain (**e**, **f**) revealed homologues populations of resting microglia cells (young, **e**; aged, **f**)). GFAP Immunoreactivity was slightly decreased in aged animals (**h**), but spatial distribution (layer 1, surrounding vessels) was similar (young: **g**; aged: **h**). Campbell-Switzer stain unveiled neither extracellular plaques nor tangles (**i**, **j**). Thioflavin T likewise indicated no amyloid plaques (young, **k**; aged, **l**). Semi-automatically determination of neurons (**m**) as well as microglial cells (**n**) and astrocytes (**o**) in cortices revealed no significant changes in aged animals. Scale bars = 100 μm. Data is presented as mean ± SEM (n ≥ 4)

Routine histological Haematoxylin and Eosin (H&E) stains of young (1 year) and old (5 years) wild-type degus revealed normal age-related changes in the examined brain regions, but no obvious signs for specific lesions, neurodegeneration, or neuronal displacement (Fig. 2a, b). Neuronal marker NeuN unveiled no generalized loss of cortical neurons between both time points (Fig. 2c, d, m). Microglia-specific stains of ionized calcium-binding adapter molecule 1 (IBA1), which can unravel early signs of pathology shown by localised microglia accumulation, displayed homogenously distributed populations of resting microglia in young and aged animals (Fig. 2e, f). Individual differences in young and aged degus were apparent, but the cortical coverage was not significantly different between the two groups (Fig. 2n). Cortical clustering of microglia as seen in pre-depositing APP-transgenic mice, pinpointing towards starting Aβ deposition, was not detected. Cortical astroglia (GFAP-positive) were nearly exclusively located in cortical layer 1 and around blood vessels (Fig. 2g, h, o), without any age-dependent changes in spatial distribution or intensity.

To evaluate the extent of any cortical amyloidosis and neuronal destruction, a modified Campbell-Switzer stain was applied, but no extracellular deposits were exposed (Fig. 2i, j). This result was supported by thioflavin T stains which revealed no specific cortical fluorescence as well (Fig. 2k, l).

**Fig. 3:**
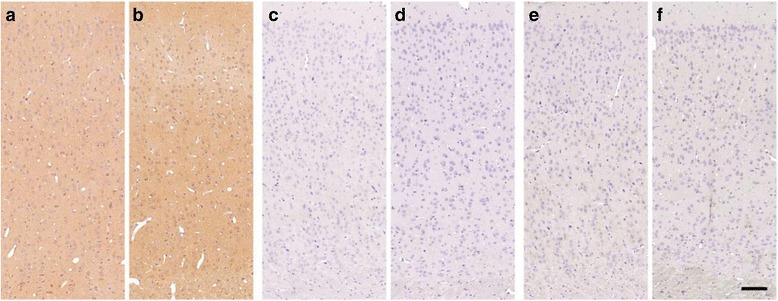
β-amyloid pathology in young and aged degus. Aβ staining using 6E10-antibody (**a**, **b**) resulted in unspecific background signals accompanied by spot-like intracellular immunoreactivity. Although visible in both, young (**a**) and aged (**b**), intensity generally tends to be elevated in aged animals. In contrast, no immunoreactivity was detected in young (**c**, **e**) or aged (**d**, **f**) degus using 6F3D (**c**, **d**), or 4G8 (**e**, **f**). Scale bar = 100 μm

To examine potentially undiscovered amyloid deposits, we performed more sensitive immunohistochemical stains by employing commonly used antibodies against different Aβ epitopes (clones 6F3D, 4G8, 6E10). The epitope of clone 6E10 is located N-terminal of the H13R substitution (within amino acids 3-8). Besides high unspecific background staining, limited intracellular immunoreactivity was detected in all cortices of young and aged degus. However, extracellular deposits (e.g. plaques) could not be traced in any of the examined brain regions (Fig. 3a, b). The epitope of anti-Aβ antibody clone 6F3D (amino acids 8-17) includes the H13R substitution and showed neither intra- nor extracellular immunoreactivity in young or aged animals (Fig. 3c, d). Likewise, no aggregates could be detected using the 4G8 antibody (Fig. 3e, f) with an epitope C-terminally of the H13R substitution (amino acids: 18-22). The lack of age-dependent, immunohistological changes in degus was independently confirmed by a second neuropathological laboratory (C.K.) in an additional study (1 to 5 year old degus from the same colony, anti-amyloid stains using clones 6E10 and IC16; data not shown).

**Fig. 4:**
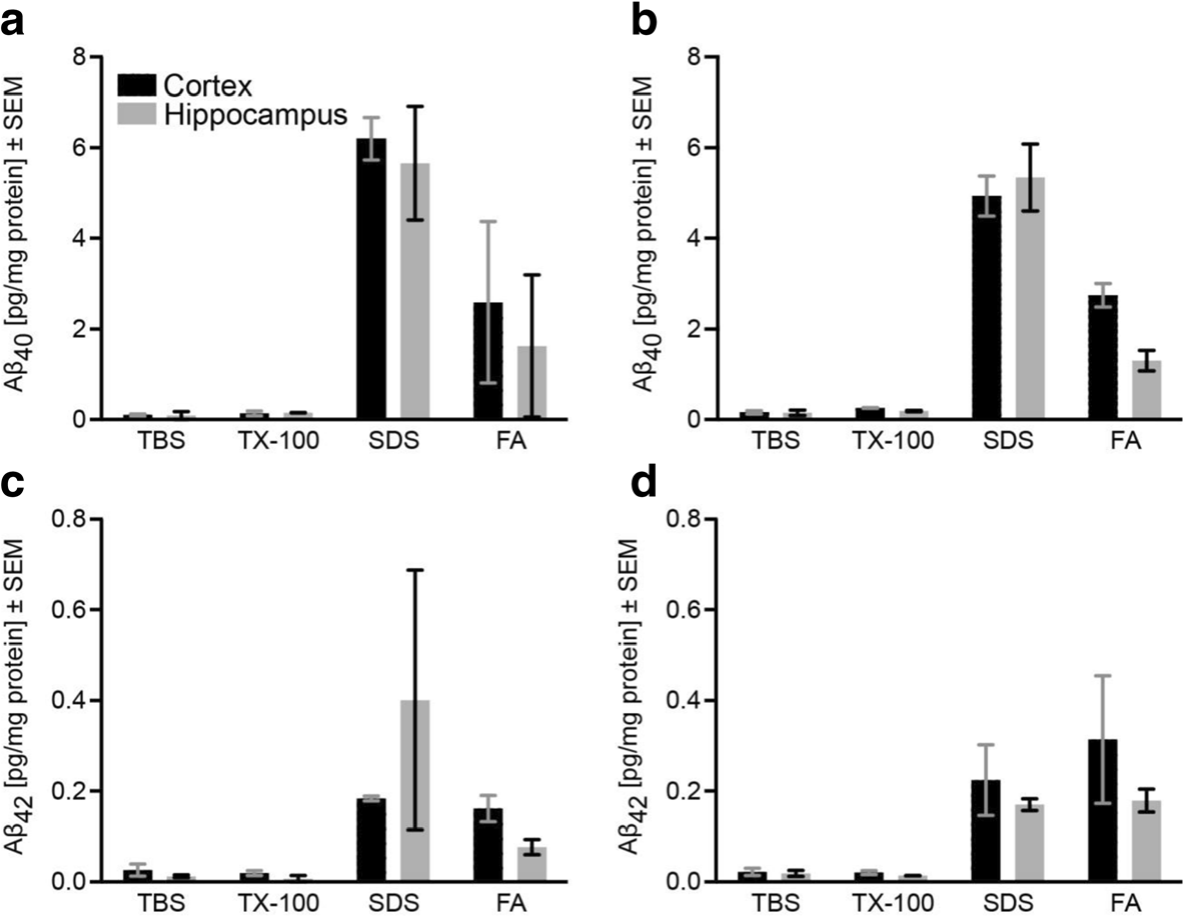
Aβ levels in fractionated brain tissue of young and aged degus. Levels of Aβ_40_ and Aβ_42_ were measured in fractionated cortical (*black*) and hippocampal (*grey*) tissue of young (**a**, **c**) and aged (**b**, **d**) degus using immunoassays. **a**, **b** In both groups, Aβ_40_ was rarely present in soluble (TBS) and membrane-bound (TX-100) forms. The highest amounts were protein-bound (SDS) and smaller proportions were insoluble (formic acid; FA). Overall, young and aged animals demonstrated very similar levels Aβ_40_. **c**, **d** Young and aged degus showed low levels of Aβ_42_ in soluble (TBS) and membrane-bound (TX-100) fractions and higher levels in protein-bound (SDS) and insoluble (FA) fractions in both, cortex and hippocampus. Aβ_42_ levels were likewise not crucially changed in aged animals. Data is presented as mean ± SEM (young: 3, 24 months; aged: 56, 56, 65, 65 months)

**Fig. 5:**
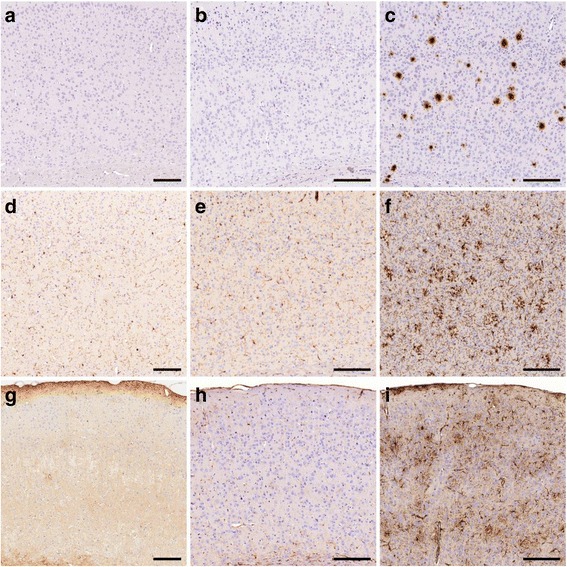
Comparison of neuropathological changes in wild-type degus, wild-type and transgenic mice. While aged wild-type degus (**a**, 5 years old) and mice (**b**, 200-days-old) exhibit no sign of β-amyloid deposition, APP/PS1 transgenic mice present with obvious cortical amyloidosis (**c**, 150-days-old). Compared to wild-type degus (**d**, **g**) and mice (**e**, **h**), transgenic mice manifest with pronounced micro- (**f**) and astrogliosis (**i**). Scale bars = 200 μm

Levels of cortical and hippocampal Aβ_40_ and Aβ_42_ in young and aged degus were quantitatively measured using immunoassays (Fig. 4), revealing very low levels of soluble and membrane-bound Aβ and low levels of protein-bound and insoluble Aβ. The levels of insoluble Aβ did not age-dependently change. However, the concentration of insoluble Aβ was generally several magnitudes below those of established AD mouse models, and even lower than those of wild-type naked mole rats and guinea pigs (Table 2). The comparison of wild-type degus, wild-type and transgenic mice demonstrates that aged wild-type degus very closely resemble histological parameters of wild-type mice in terms of Aβ deposition and unspecific activation of microglial and astroglial cells (Fig. 5).

**Table 2:** Levels of insoluble Aβ in transgenic AD models and wild-type rodents

	Model	Age (months)	Aβ_40_ (pg/mg)	Aβ_42_ (pg/mg)	Reference
AD models	APP23	12	3098	746	[54]
	APP_London_	24	1300	3300	[55]
	Tg2576	2–3	700	2100	[56]
		>20	39,900	40,900	[57]
	APP/PS1	5	26,200	49,400	[58]
		8	166,000	113,500	
Wild-type naked mole rats		2–9 years	37	60	[34]
Guinea pig		adult	79	18	[59]

**Fig. 6:**
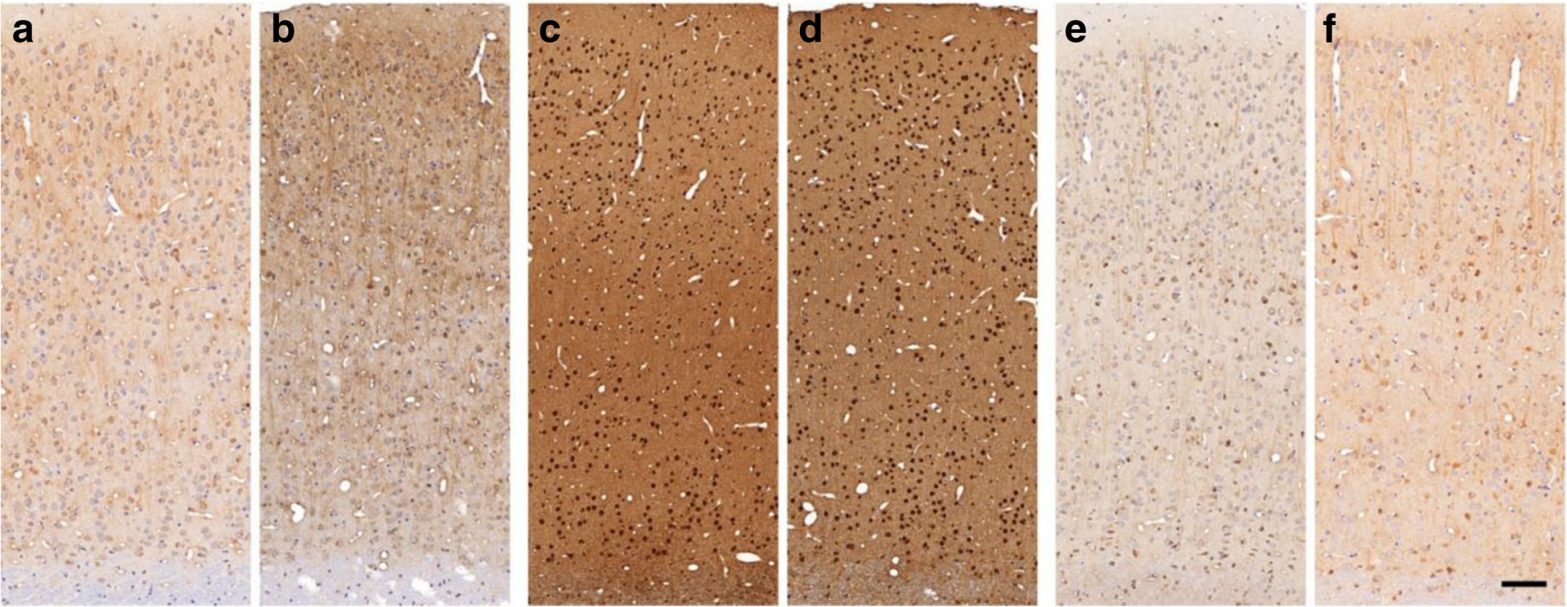
Tau pathology in young and aged degus. Phosphoepitope-specific antibodies were used to examine involvement of tau. AT8 revealed intracellular immunoreactivity in young and aged degus (**a**, **b**). AT100 was unspecific and appeared almost entirely in the nucleus (**c**, **d**). AT180-labelled cortical neurons in young and aged animals (**e**, **f**). Scale bar = 100 μm

Phosphorylated tau is the second protein accumulating during disease progression and another histopathological hallmark of AD [31]. We utilized antibodies against different epitopes of phosphorylated tau to screen for neurofibrillary tangles. Although sequences of human and degu tau vary, the analysed phosphorylation sites (Ser202/Thr205, Thr212/Ser214 and Thr231) are identical (Fig. 1c). AT8 (Ser202/Thr205) labelled cortical neurons in young and aged animals (Fig. 6a, b) and AT100 (Thr212/Ser214) showed nuclear-localized reactivity (Fig. 6c, d). AT100 epitope is known for nuclear co-localization and considered not tau-specific, as it appears likewise in tau knockout mice [32]. AT180 (Thr231) equally stained cortical neurons of young and aged degus (Fig. 6e, f). In sum, the detected tau did morphologically not correspond to neurofibrillary tangles and showed no age-dependent intensification.

**Fig. 7:**
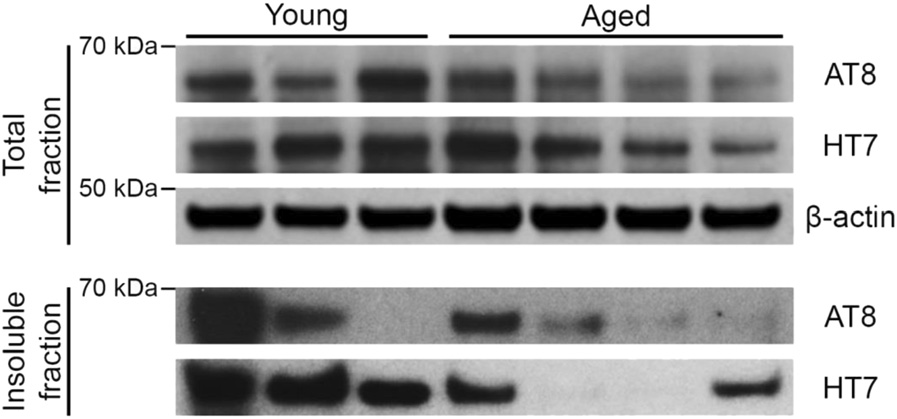
Total and insoluble levels of cortical tau in young and aged degus. Biochemical analysis of total and insoluble tau in cortices of young and aged degus revealed high individual variance, but no significant age-related changes in levels of neither total (HT7) nor phosphorylated (AT8) tau. β-actin served as loading control. Age of degus (left to right in months): 3, 24, 25, 56, 56, 65, 65

Moreover, the independent quantification of tau revealed neither elevated levels of total tau, nor an increase in phosphorylated or insoluble fraction (Fig. 7).

## Discussion

In the near future, aging societies will be particularly challenged by age-related diseases demanding intensive care. Exceptional research endeavours are necessary to face these approaching challenges. Thus, reliable and accurate animal models are one of the major prerequisites in basic research. Although transgenic rodent models of AD are constantly refined, no model yet mimics all pathological features of this complex disease. More accurate models maintaining the beneficial characteristics of rodents would lead to a better understanding and more expedient therapeutic approaches. Degus were described as a promising natural model of Alzheimer’s disease during the last years by a Chilean group. However, in our studies we were unable to detect any systematic occurrence of the typical histopathological hallmarks of AD in relation to age. Haematoxylin and Eosin as well as NeuN stains showed not more than slight differences between young and aged degus, rather indicative for the natural aging process than pathological neurodegeneration. In contrast to previous results from Inestrosa et al. [15], we could not detect elevated GFAP expression in old degus compared to young animals. The lack of signs for microglial and astrocytic inflammation further reiterates the absence of a pathological degeneration in the examined brains of the old degus.

### Aβ pathology

The sparse intracellular reactivity of anti-Aβ clone 6E10, which was lacking in clone 4G8, clone 6F3D, Campbell-Switzer and thioflavin T stains, most likely indicates an unspecific reaction [33]. In contrast, no sign of any *extracellular* deposition of Aβ was detected in aged animals by any of the applied staining methods (Fig. 3). Quantitative measurements underpinned the absence of considerable amounts of insoluble Aβ (Fig. 4) and revealed Aβ-levels that are in the same range as those in wild-type mice [29] and below those of wild-type naked mole rats [34].

Consistent with results of van Groen et al. no significant neuronal loss was found in the brain of 5-years-old degus [13]. These findings are in sharp contrast to observations in brains of degus obtained from their natural habitat, in which prominent intra- and extracellular Aβ deposits in cortices and hippocampi of aged animals (>3 years) were reported [12, 19]. These differences may, at least in part, be caused by different rearing conditions (laboratory housing versus natural wildlife conditions) and it has to be considered that in their wildlife habitat the animals are exposed to stress, may suffer from hypertension, viral infections and diabetes, i.e. known risk factors contributing to the aetiology of AD and the early development of AD-type neuropathology.

Furthermore, the single amino-acid-difference between degus and humans at position 13 (histidine to arginine) affects a histidine residue (His^13^) which is crucial for aggregation and toxicity of Aβ. His^13^ is involved in early N-terminal β-sheet formation [35] and a substitution lowers aggregation propensity [36], neuronal binding [37], and cytotoxicity [36]. Moreover, His^13^ is involved in the coordination of metal ions [38] and methylation or substitution by arginine, as seen in degus, lowers the affinity for metal ions and thus depletes aggregation [38–40] and attenuates toxicity [41, 42] of Aβ.

Two other species which are related to degus share a similar Aβ sequence, but, despite higher life expectancies, lack the neuropathological features as reported for degus. Naked mole rats (*Heterocephalus glaber*) have the identical Aβ sequence (see Fig. 1) and an exceptional lifespan of more than 30 years [34]. Although young naked mole rats naturally exhibit pronounced oxidative stress [43] and *Aβ levels similar to 3xTg-AD* mice [34, 43], they *do not develop amyloid plaques* with age [34]. Furthermore, naked mole rats even present with high levels of phosphorylated tau without any tangle formation [44]. In Guinea pigs (*Cavia porcellus*), with a human *identical* Aβ sequence (see Fig. 1) and a lifespan similar to degus (average 5–7 [45]), dense amyloid deposits do not occur [45], despite similar APP processing [46, 47] and high β–secretase activity [47].

### Tau pathology

The additional screening for tau deposition, the second aggregating protein in AD, revealed similar intracellular reactivity in young and aged degus using phosphoepitope-specific antibodies AT8 and AT180. AT100 staining showed the previously described, unspecific nuclear localization [32]. Biochemical analysis did not reveal an age-dependent increase of total, insoluble or phosphorylated tau (Fig. 7). Some variability observed in the levels of total tau or insoluble tau could hint subsets with different aggregation propensities but the very same animals did not exhibit tau pathology in IHC, and larger number of animals would be needed to identify the existence of such subsets. Hence, no evidence for a pathological deposition of tau could be detected in the examined animals.

### Methodological considerations

The animals used in a variety of studies were collected from different sources [13, 48, 49], including animals caught in the wild [12, 50], the latter does usually not allow a precise age determination and thus hinders precisely controlled analyses. However, standardised housing conditions as used for the degus examined in the present study seem to prevent the development of ‘AD-like’ pathology described for wild-caught animals. Nonetheless, it would be fascinating to decipher the factors inducing the histopathological and biochemical changes in degus previously described [12, 13, 15]. Moreover, not only the particular species or the specific amino acid sequence seems influence the extent of amyloid deposition, but the genetic background likewise enfolds a strong effect [26]. As degus are not yet an established research model, they lack a defined, stable and characterized inbred genetic background.

An interesting approach of separating old degus with severely aberrant behaviour as disease model takes high individual variability into account and indicates that ‘AD-like’ pathology might not necessarily develop in old degus. However, even in this selected subgroup with increased levels of inflammatory and oxidative stress markers, no correlation between altered behaviour and specific neuropathological symptoms could be established [50]. The stated impairments of spatial memory and cognition in aged degus [14] may therefore be just a part of the normal aging process, since physiologic aging is linked to significant impairments in memory [51], cognition [52] and hippocampal long-term potentiation [53] in mice as well. Thus, symptoms of normal aging may not be misinterpreted to model AD.

## Conclusion

*Octodon degus* was re-evaluated in the context of existing rodent AD models and human AD pathology. Performing immunohistological and molecular analyses of young and aged animals, we were able to show exclusively normal age-related cortical changes without indications for extensive degeneration as seen in patients with dementia and transgenic AD mice. Neither significant neuronal loss nor enhanced microglial activation were observed in aged animals of our degu population. Furthermore, no amyloid accumulation or tangle formation as seen in sporadic Alzheimer’s disease patients could be determined. Phosphoepitope-specific antibodies against tau species displayed similar intracellular neuronal reactivity in both, young and aged degus.

Moreover, we highlighted some previous results, which stand in contrast to the assumption of degus as natural AD model and seem to be thus far neglected. Bearing that in mind, assessment of degus as AD models should be meticulously done and receive particular attention. Currently, it is not clear if unnoticed environmental or rearing factors might play a role in triggering AD-like neuropathology. Therefore, we conclude that presently, the rodent *Octodon degus* is neither a superior model which is more suitable than other frequently used rodent models nor a ‘natural’ model of Alzheimer’s disease in general.

## References

[1] Glabe CG (2006). Common mechanisms of amyloid oligomer pathogenesis in degenerative disease. Neurobiol Aging.

[2] Kayed R, Lasagna-Reeves CA (2013). Molecular mechanisms of amyloid oligomers toxicity. J Alzheimers Dis.

[3] Choi SH, Kim YH, Hebisch M, Sliwinski C, Lee S, D'Avanzo C, Chen H, Hooli B, Asselin C, Muffat J, Klee JB, Zhang C, Wainger BJ, Peitz M, Kovacs DM, Woolf CJ, Wagner SL, Tanzi RE, Kim DY (2014). A three-dimensional human neural cell culture model of Alzheimer's disease. Nature.

[4] Pflanzner T, Janko MC, Andre-Dohmen B, Reuss S, Weggen S, Roebroek AJ, Kuhlmann CR, Pietrzik CU (2011). LRP1 mediates bidirectional transcytosis of amyloid-beta across the blood-brain barrier. Neurobiol Aging.

[5] Krohn M, Lange C, Hofrichter J, Scheffler K, Stenzel J, Steffen J, Schumacher T, Bruning T, Plath AS, Alfen F, Schmidt A, Winter F, Rateitschak K, Wree A, Gsponer J, Walker LC, Pahnke J (2011). Cerebral amyloid-beta proteostasis is regulated by the membrane transport protein ABCC1 in mice. J Clin Invest.

[6] Pahnke J, Frohlich C, Paarmann K, Krohn M, Bogdanovic N, Arsland D, Winblad B (2014). Cerebral ABC transporter-common mechanisms may modulate neurodegenerative diseases and depression in elderly subjects. Arch Med Res.

[7] Reitz C, Mayeux R (2014). Alzheimer disease: epidemiology, diagnostic criteria, risk factors and biomarkers. Biochem Pharmacol.

[8] Hollingworth P, Harold D, Sims R, Gerrish A, Lambert JC, Carrasquillo MM, Abraham R, Hamshere ML, Pahwa JS, Moskvina V, Dowzell K, Jones N, Stretton A, Thomas C, Richards A, Ivanov D, Widdowson C, Chapman J, Lovestone S, Powell J, Proitsi P, Lupton MK, Brayne C, Rubinsztein DC, Gill M, Lawlor B, Lynch A, Brown KS, Passmore PA, Craig D, McGuinness B, Todd S, Holmes C, Mann D, Smith AD, Beaumont H, Warden D, Wilcock G, Love S, Kehoe PG, Hooper NM, Vardy ER, Hardy J, Mead S, Fox NC, Rossor M, Collinge J, Maier W, Jessen F, Ruther E, Schurmann B, Heun R, Kolsch H, van den Bussche H, Heuser I, Kornhuber J, Wiltfang J, Dichgans M, Frolich L, Hampel H, Gallacher J, Hull M, Rujescu D, Giegling I, Goate AM, Kauwe JS, Cruchaga C, Nowotny P, Morris JC, Mayo K, Sleegers K, Bettens K, Engelborghs S, De Deyn PP, Van Broeckhoven C, Livingston G, Bass NJ, Gurling H, McQuillin A, Gwilliam R, Deloukas P, Al-Chalabi A, Shaw CE, Tsolaki M, Singleton AB, Guerreiro R, Muhleisen TW, Nothen MM, Moebus S, Jockel KH, Klopp N, Wichmann HE, Pankratz VS, Sando SB, Aasly JO, Barcikowska M, Wszolek ZK, Dickson DW, Graff-Radford NR, Petersen RC, Alzheimer's Disease Neuroimaging I, van Duijn CM, Breteler MM, Ikram MA, DeStefano AL, Fitzpatrick AL, Lopez O, Launer LJ, Seshadri S, Consortium C, Berr C, Campion D, Epelbaum J, Dartigues JF, Tzourio C, Alperovitch A, Lathrop M, Consortium E, Feulner TM, Friedrich P, Riehle C, Krawczak M, Schreiber S, Mayhaus M, Nicolhaus S, Wagenpfeil S, Steinberg S, Stefansson H, Stefansson K, Snaedal J, Bjornsson S, Jonsson PV, Chouraki V, Genier-Boley B, Hiltunen M, Soininen H, Combarros O, Zelenika D, Delepine M, Bullido MJ, Pasquier F, Mateo I, Frank-Garcia A, Porcellini E, Hanon O, Coto E, Alvarez V, Bosco P, Siciliano G, Mancuso M, Panza F, Solfrizzi V, Nacmias B, Sorbi S, Bossu P, Piccardi P, Arosio B, Annoni G, Seripa D, Pilotto A, Scarpini E, Galimberti D, Brice A, Hannequin D, Licastro F, Jones L, Holmans PA, Jonsson T, Riemenschneider M, Morgan K, Younkin SG, Owen MJ, O'Donovan M, Amouyel P, Williams J (2011). Common variants at ABCA7, MS4A6A/MS4A4E, EPHA1, CD33 and CD2AP are associated with Alzheimer's disease. Nat Genet.

[9] Schellenberg GD, Montine TJ (2012). The genetics and neuropathology of Alzheimer's disease. Acta Neuropathol.

[10] Braidy N, Munoz P, Palacios AG, Castellano-Gonzalez G, Inestrosa NC, Chung RS, Sachdev P, Guillemin GJ (2012). Recent rodent models for Alzheimer's disease: clinical implications and basic research. J Neural Transm (Vienna).

[11] Ebensperger LA, Tapia D, Ramirez-Estrada J, Leon C, Soto-Gamboa M, Hayes LD (2013). Fecal cortisol levels predict breeding but not survival of females in the short-lived rodent, Octodon degus. Gen Comp Endocrinol.

[12] Inestrosa NC, Reyes AE, Chacon MA, Cerpa W, Villalon A, Montiel J, Merabachvili G, Aldunate R, Bozinovic F, Aboitiz F (2005). Human-like rodent amyloid-beta-peptide determines Alzheimer pathology in aged wild-type Octodon degu. Neurobiol Aging.

[13] van Groen T, Kadish I, Popovic N, Popovic M, Caballero-Bleda M, Bano-Otalora B, Vivanco P, Rol MA, Madrid JA (2011). Age-related brain pathology in Octodon degu: blood vessel, white matter and Alzheimer-like pathology. Neurobiol Aging.

[14] Ardiles AO, Tapia-Rojas CC, Mandal M, Alexandre F, Kirkwood A, Inestrosa NC, Palacios AG (2012). Postsynaptic dysfunction is associated with spatial and object recognition memory loss in a natural model of Alzheimer's disease. Proc Natl Acad Sci U S A.

[15] Inestrosa NC, Rios JA, Cisternas P, Tapia-Rojas C, Rivera DS, Braidy N, Zolezzi JM, Godoy JA, Carvajal FJ, Ardiles AO, Bozinovic F, Palacios AG, Sachdev PS (2015). Age Progression of Neuropathological Markers in the Brain of the Chilean Rodent Octodon degus, a Natural Model of Alzheimer's Disease. Brain Pathol.

[16] Ardiles AO, Ewer J, Acosta ML, Kirkwood A, Martinez AD, Ebensperger LA, Bozinovic F, Lee TM, Palacios AG (2013). Octodon degus (Molina 1782): a model in comparative biology and biomedicine. Cold Spring Harb Protoc.

[17] Tarragon E, Lopez D, Estrada C, Ana GC, Schenker E, Pifferi F, Bordet R, Richardson JC, Herrero MT (2013). Octodon degus: a model for the cognitive impairment associated with Alzheimer's disease. CNS Neurosci Ther.

[18] Iqbal K, Bolognin S, Wang X, Basurto-Islas G, Blanchard J, Tung YC (2013). Animal models of the sporadic form of Alzheimer's disease: focus on the disease and not just the lesions. J Alzheimers Dis.

[19] Rios JA, Cisternas P, Arrese M, Barja S, Inestrosa NC (2014). Is Alzheimer's disease related to metabolic syndrome? A Wnt signaling conundrum. Prog Neurobiol.

[20] Castro-Fuentes R, Socas-Perez R (2013). Octodon degus: a strong attractor for Alzheimer research. Basic Clin Neurosci.

[21] Braidy N, Poljak A, Jayasena T, Mansour H, Inestrosa NC, Sachdev PS (2015). Accelerating Alzheimer's research through 'natural' animal models. Curr Opin Psychiatry.

[22] Rivera DS, Inestrosa NC, Bozinovic F (2016). On cognitive ecology and the environmental factors that promote Alzheimer disease: lessons from Octodon degus (Rodentia: Octodontidae). Biol Res.

[23] Radde R, Bolmont T, Kaeser SA, Coomaraswamy J, Lindau D, Stoltze L, Calhoun ME, Jaggi F, Wolburg H, Gengler S, Haass C, Ghetti B, Czech C, Holscher C, Mathews PM, Jucker M (2006). Abeta42-driven cerebral amyloidosis in transgenic mice reveals early and robust pathology. EMBO Rep.

[24] Altschul SF, Madden TL, Schaffer AA, Zhang J, Zhang Z, Miller W, Lipman DJ (1997). Gapped BLAST and PSI-BLAST: a new generation of protein database search programs. Nucleic Acids Res.

[25] Altschul SF, Wootton JC, Gertz EM, Agarwala R, Morgulis A, Schaffer AA, Yu YK (2005). Protein database searches using compositionally adjusted substitution matrices. FEBS J.

[26] Frohlich C, Paarmann K, Steffen J, Stenzel J, Krohn M, Heinze HJ, Pahnke J (2013). Genomic background-related activation of microglia and reduced beta-amyloidosis in a mouse model of Alzheimer's disease. Eur J Microbiol Immunol (Bp).

[27] Schumacher T, Krohn M, Hofrichter J, Lange C, Stenzel J, Steffen J, Dunkelmann T, Paarmann K, Frohlich C, Uecker A, Plath AS, Sommer A, Bruning T, Heinze HJ, Pahnke J (2012). ABC transporters B1, C1 and G2 differentially regulate neuroregeneration in mice. PLoS One.

[28] Frohlich C, Zschiebsch K, Groger V, Paarmann K, Steffen J, Thurm C, Schropp EM, Bruning T, Gellerich F, Radloff M, Schwabe R, Lachmann I, Krohn M, Ibrahim S, Pahnke J (2016). Activation of Mitochondrial Complex II-Dependent Respiration Is Beneficial for alpha-Synucleinopathies. Mol Neurobiol.

[29] Muller-Schiffmann A, Herring A, Abdel-Hafiz L, Chepkova AN, Schable S, Wedel D, Horn AH, Sticht H, de Souza Silva MA, Gottmann K, Sergeeva OA, Huston JP, Keyvani K, Korth C (2016). Amyloid-beta dimers in the absence of plaque pathology impair learning and synaptic plasticity. Brain.

[30] Oshima N, Morishima-Kawashima M, Yamaguchi H, Yoshimura M, Sugihara S, Khan K, Games D, Schenk D, Ihara Y (2001). Accumulation of amyloid beta-protein in the low-density membrane domain accurately reflects the extent of beta-amyloid deposition in the brain. Am J Pathol.

[31] Grundke-Iqbal I, Iqbal K, Quinlan M, Tung YC, Zaidi MS, Wisniewski HM (1986). Microtubule-associated protein tau. A component of Alzheimer paired helical filaments. J Biol Chem.

[32] Whiteman IT, Minamide LS, de Goh L, Bamburg JR, Goldsbury C (2011). Rapid changes in phospho-MAP/tau epitopes during neuronal stress: cofilin-actin rods primarily recruit microtubule binding domain epitopes. PLoS One.

[33] Aho L, Pikkarainen M, Hiltunen M, Leinonen V, Alafuzoff I (2010). Immunohistochemical visualization of amyloid-beta protein precursor and amyloid-beta in extra- and intracellular compartments in the human brain. J Alzheimers Dis.

[34] Edrey YH, Medina DX, Gaczynska M, Osmulski PA, Oddo S, Caccamo A, Buffenstein R (2013). Amyloid beta and the longest-lived rodent: the naked mole-rat as a model for natural protection from Alzheimer's disease. Neurobiol Aging.

[35] Dong X, Chen W, Mousseau N, Derreumaux P (2008). Energy landscapes of the monomer and dimer of the Alzheimer's peptide Abeta(1-28). J Chem Phys.

[36] Dai X, Sun Y, Gao Z, Jiang Z (2010). Copper enhances amyloid-beta peptide neurotoxicity and non beta-aggregation: a series of experiments conducted upon copper-bound and copper-free amyloid-beta peptide. J Mol Neurosci.

[37] Poduslo JF, Howell KG, Olson NC, Ramirez-Alvarado M, Kandimalla KK (2012). Alzheimer's disease amyloid beta-protein mutations and deletions that define neuronal binding/internalization as early stage nonfibrillar/fibrillar aggregates and late stage fibrils. Biochemistry.

[38] Liu ST, Howlett G, Barrow CJ (1999). Histidine-13 is a crucial residue in the zinc ion-induced aggregation of the A beta peptide of Alzheimer's disease. Biochemistry.

[39] Huang J, Yao Y, Lin J, Ye YH, Sun WY, Tang Dagger WX (2004). The solution structure of rat Abeta-(1-28) and its interaction with zinc ion: insights into the scarcity of amyloid deposition in aged rat brain. J Biol Inorg Chem.

[40] Miura T, Suzuki K, Kohata N, Takeuchi H (2000). Metal binding modes of Alzheimer's amyloid beta-peptide in insoluble aggregates and soluble complexes. Biochemistry.

[41] Smith DP, Smith DG, Curtain CC, Boas JF, Pilbrow JR, Ciccotosto GD, Lau TL, Tew DJ, Perez K, Wade JD, Bush AI, Drew SC, Separovic F, Masters CL, Cappai R, Barnham KJ (2006). Copper-mediated amyloid-beta toxicity is associated with an intermolecular histidine bridge. J Biol Chem.

[42] Tickler AK, Smith DG, Ciccotosto GD, Tew DJ, Curtain CC, Carrington D, Masters CL, Bush AI, Cherny RA, Cappai R, Wade JD, Barnham KJ (2005). Methylation of the imidazole side chains of the Alzheimer disease amyloid-beta peptide results in abolition of superoxide dismutase-like structures and inhibition of neurotoxicity. J Biol Chem.

[43] Edrey YH, Oddo S, Cornelius C, Caccamo A, Calabrese V, Buffenstein R (2014). Oxidative damage and amyloid-beta metabolism in brain regions of the longest-lived rodents. J Neurosci Res.

[44] Orr ME, Garbarino VR, Salinas A, Buffenstein R (2015). Sustained high levels of neuroprotective, high molecular weight, phosphorylated tau in the longest-lived rodent. Neurobiol Aging.

[45] Bates K, Vink R, Martins R, Harvey A (2014). Aging, cortical injury and Alzheimer's disease-like pathology in the guinea pig brain. Neurobiol Aging.

[46] Beck M, Bruckner MK, Holzer M, Kaap S, Pannicke T, Arendt T, Bigl V (2000). Guinea-pig primary cell cultures provide a model to study expression and amyloidogenic processing of endogenous amyloid precursor protein. Neuroscience.

[47] Beck M, Bigl V, Rossner S (2003). Guinea pigs as a nontransgenic model for APP processing in vitro and in vivo. Neurochem Res.

[48] Palacios-Munoz A, Escobar MJ, Vielma A, Araya J, Astudillo A, Valdivia G, Garcia IE, Hurtado J, Schmachtenberg O, Martinez AD, Palacios AG (2014). Role of connexin channels in the retinal light response of a diurnal rodent. Front Cell Neurosci.

[49] Kumazawa-Manita N, Katayama M, Hashikawa T, Iriki A (2013). Three-dimensional reconstruction of brain structures of the rodent Octodon degus: a brain atlas constructed by combining histological and magnetic resonance images. Exp Brain Res.

[50] Deacon RM, Altimiras FJ, Bazan-Leon EA, Pyarasani RD, Nachtigall FM, Santos LS, Tsolaki AG, Pednekar L, Kishore U, Biekofsky RR, Vasquez RA, Cogram P (2015). Natural AD-Like Neuropathology in Octodon degus: Impaired Burrowing and Neuroinflammation. Curr Alzheimer Res.

[51] Li Q, Zhao HF, Zhang ZF, Liu ZG, Pei XR, Wang JB, Cai MY, Li Y (2009). Long-term administration of green tea catechins prevents age-related spatial learning and memory decline in C57BL/6 J mice by regulating hippocampal cyclic amp-response element binding protein signaling cascade. Neuroscience.

[52] Palmeri A, Privitera L, Giunta S, Loreto C, Puzzo D (2013). Inhibition of phosphodiesterase-5 rescues age-related impairment of synaptic plasticity and memory. Behav Brain Res.

[53] Puzzo D, Bizzoca A, Loreto C, Guida CA, Gulisano W, Frasca G, Bellomo M, Castorina S, Gennarini G, Palmeri A (2015). Role of F3/contactin expression profile in synaptic plasticity and memory in aged mice. Neurobiol Aging.

[54] He P, Cheng X, Staufenbiel M, Li R, Shen Y (2013). Long-term treatment of thalidomide ameliorates amyloid-like pathology through inhibition of beta-secretase in a mouse model of Alzheimer's disease. PLoS One.

[55] Pype S, Moechars D, Dillen L, Mercken M (2003). Characterization of amyloid beta peptides from brain extracts of transgenic mice overexpressing the London mutant of human amyloid precursor protein. J Neurochem.

[56] Toda T, Noda Y, Ito G, Maeda M, Shimizu T (2011). Presenilin-2 mutation causes early amyloid accumulation and memory impairment in a transgenic mouse model of Alzheimer's disease. J Biomed Biotechnol.

[57] Parkinson J, Ploeger B, Appelkvist P, Bogstedt A, Dillner Bergstedt K, Eketjall S, Visser SA (2013). Modeling of age-dependent amyloid accumulation and gamma-secretase inhibition of soluble and insoluble Abeta in a transgenic mouse model of amyloid deposition. Pharmacol Res Perspect.

[58] Krauthausen M, Kummer MP, Zimmermann J, Reyes-Irisarri E, Terwel D, Bulic B, Heneka MT, Muller M (2015). CXCR3 promotes plaque formation and behavioral deficits in an Alzheimer's disease model. J Clin Invest.

[59] Beach TG, Kuo YM, Schwab C, Walker DG, Roher AE (2001). Reduction of cortical amyloid beta levels in guinea pig brain after systemic administration of physostigmine. Neurosci Lett.

